# Evaluation of ER, PgR, HER-2 and Ki-67 as predictors of response to neoadjuvant anthracycline chemotherapy for operable breast cancer

**DOI:** 10.1038/sj.bjc.6602256

**Published:** 2004-12-21

**Authors:** R J Burcombe, A Makris, P I Richman, F M Daley, S Noble, M Pittam, D Wright, S A Allen, J Dove, G D Wilson

**Affiliations:** 1 Academic Oncology Unit, Mount Vernon Hospital, Rickmansworth Road, Northwood, Middlesex HA6 2RN, UK; 2 Gray Cancer Institute, Mount Vernon Hospital, Rickmansworth Road, Northwood, Middlesex HA6 2RN, UK; 3 Luton & Dunstable Hospital, Lewsey Road, Luton, Bedfordshire LU4 0DZ, UK

**Keywords:** breast cancer, chemotherapy response, ER, HER-2/neu, Ki-67, neoadjuvant chemotherapy, PgR

## Abstract

Primary systemic therapy (PST) for operable breast cancer enables the identification of *in vivo* biological markers that predict response to treatment. A total of 118 patients with T2–4 N0–1 M0 primary breast cancer received six cycles of anthracycline-based PST. Clinical and radiological response was assessed before and after treatment using UICC criteria. A grading system to score pathological response was devised. Diagnostic biopsies and postchemotherapy surgical specimens were stained for oestrogen (ER) and progesterone (PgR) receptor, HER-2 and cell proliferation (Ki-67). Clinical, radiological and pathological response rates were 78, 72 and 38%, respectively. There was a strong correlation between ER and PgR staining (*P*<0.0001). Higher Ki-67 proliferation indices were associated with PgR− tumours (median 28.3%, PgR+ 22.9%; *P*=0.042). There was no relationship between HER-2 and other biological markers. No single pretreatment or postchemotherapy biological parameter predicted response by any modality of assessment. In all, 10 tumours changed hormone receptor classification after chemotherapy (three ER, seven PgR); HER-2 staining changed in nine cases. Median Ki-67 index was 24.9% before and 18.1% after treatment (*P*=0.02); the median reduction in Ki-67 index after treatment was 21.2%. Tumours displaying >75% reduction in Ki-67 after chemotherapy were more likely to achieve a pathological response (77.8 *vs* 26.7%, *P*=0.004).

Neoadjuvant chemotherapy or primary systemic therapy (PST) for operable breast cancer provides an opportunity to evaluate biological markers that may predict response to treatment. The identification of tumour parameters that accurately predict response to treatment would be of value in optimising PST so that nonresponders could be offered alternative and more effective therapies.

Biological markers used in routine clinical practice were selected for the purposes of this analysis. Oestrogen receptor (ER) and progesterone receptor (PgR) status are widely used to select patients for adjuvant hormonal therapy (Allred *et al*, 1998; EBCTCG, 1998; Harvey *et al*, 1999) and may predict response to cytotoxic chemotherapy in metastatic disease (Kiang *et al*, 1978; Lippman *et al*, 1978; Lippman and Allegra, 1980), but their role in predicting response to primary chemotherapy remains controversial. Human epidermal growth factor (HER-2) overexpression has been associated with poor overall prognosis, shorter relapse-free and overall survival (Slamon *et al*, 1987), sensitivity to optimal dose anthracycline chemotherapy (Thor *et al*, 1998) and resistance to adjuvant anthracycline chemotherapy and tamoxifen (Willsher *et al*, 1998; Gregory *et al*, 2000). Immunohistochemical staining for Ki-67, a nuclear antigen expressed in cycling cells, allows the proliferation index of tumours to be estimated on paraffin-embedded tissue sections. Higher responses to chemotherapy have been reported in patients with rapidly proliferating tumours (Remvikos *et al*, 1989; Bottini *et al*, 1996; Collecchi *et al*, 1998), but other studies have failed to confirm this association (Silvestrini *et al*, 1987; Bonadonna *et al*, 1990; Makris *et al*, 1997; Ozmen *et al*, 2001).

The optimum method for assessing response to neoadjuvant chemotherapy is unclear. Clinical response has traditionally been used as a surrogate intermediate end point in biological marker studies although the NSABP-B18 trial, the largest randomised trial of PST to date, demonstrated that clinical response predicted disease-free but not overall survival (Fisher *et al*, 1997). However, recent reports from The Royal Marsden Hospital show that good clinical responders (patients with complete clinical response or residual thickening only) achieve superior overall survival at 10 years compared to poor clinical responders (Chang *et al*, 1999; Cleator *et al*, 2003). Radiological assessment by mammography and/or ultrasound has been used as a more objective measurement of response, but correlation with long-term survival has not been widely studied. Complete pathological response has consistently been shown to be associated with improved outcome, but only a small minority of patients display complete tumour regression after treatment, reducing its clinical utility as a biological end point (Sataloff *et al*, 1995). Patients who achieve a complete pathological response (pCR) have a statistically significantly superior survival compared to those with residual invasive carcinoma after PST (Fisher *et al*, 1998). Scoring systems that grade incomplete pathological responses have been devised but have not been consistently applied. In this study, therefore, a scoring system devised to grade pathological response has been used alongside traditional clinical and radiological end points. In the absence of an optimal method of response assessment, we adopted a pragmatic approach to measure response by clinical, radiological and pathological criteria to evaluate whether ER, PgR, HER-2 status or Ki-67 proliferation index before or after treatment predict response to anthracycline-based PST in women with primary breast cancer.

## MATERIALS AND METHODS

### Treatment protocol

During the period September 1995–September 1999, 118 patients with primary operable (T2–T4, N0 or N1, M0) invasive primary breast carcinoma were identified (Table 1). Women with inflammatory breast cancer (T4d) or metastatic disease (M1) were excluded. All patients underwent diagnostic core biopsy of the breast tumour to confirm invasive carcinoma before commencing treatment. Bidimensional clinical tumour measurements were performed prior to every cycle of treatment. Radiological measurements of tumour size were recorded before and on completion of chemotherapy in 94 patients by mammography and/or breast ultrasound. Patients with responding or stable disease by clinical criteria received six cycles of anthracycline-based chemotherapy administered at 21-day intervals. Surgery was performed approximately 1 month after the final cycle of chemotherapy: 46 women had breast-conserving surgery, 55 required mastectomy and one patient underwent axillary dissection only. All patients managed by breast-conserving surgery received post-operative radiation to the residual breast (40 Gy in 15 daily fractions over 3 weeks plus 10 Gy boost to the tumour bed in five fractions; *n*=32); 14 patients also had the draining lymph node areas irradiated (50 Gy in 25 fractions over 5 weeks). Post-mastectomy chest wall radiation was delivered to 46 of 55 patients (32 chest wall only, 14 chest wall and lymph nodes). No patient received postoperative chemotherapy. Women with ER+ tumours received 5 years adjuvant Tamoxifen (20 mg daily) starting after surgery.

Five patients developed disease progression by clinical criteria; four proceeded to immediate surgery. The remaining patient progressed by clinical and radiological criteria after three cycles of FEC chemotherapy; she responded to four cycles of second-line docetaxel and had a near-complete pathological response at operation (1 mm residual invasive carcinoma). A group of 16 patients who attained a complete clinical and/or radiological response received radical radiotherapy to the breast and draining nodal areas, but no surgery. The local research and ethics committee approved the study prior to patient recruitment.

### Chemotherapy schedules

Patients received a maximum of six cycles of chemotherapy at 21-day intervals using one of the following regimens (see Table 1): 5-fluorouracil (5FU) 600 mg m^−2^, epirubicin 60 mg m^−2^, cyclophosphamide 600 mg m^−2^ (FEC); adriamycin 60 mg m^−2^, cyclophosphamide 600 mg m^−2^ (AC); cyclophosphamide 600 mg m^−2^, adriamycin 60 mg m^−2^, 5FU 600 mg m^−2^ (CAF); epirubicin 50 mg m^−2^, cisplatin 60 mg m^−2^; 5FU 200 mg m^−2^ day^−1^ via Hickman line (ECF) or methotrexate 30 mg m^−2^ (max 45 mg), mitoxantrone 7 mg m^−2^, mitomycin C 7 mg m^−2^ (alternate cycles only) (MMM).

### Assessment of response

#### Clinical and radiological response

Standard UICC criteria (Hayward *et al*, 1977) were used to categorise objective clinical and radiological response. Changes in the calculated product of bidimensional clinical (c) or radiological (r) tumour measurements at diagnosis and on completion of chemotherapy were recorded. Complete response (CR) was defined as no residual palpable or radiological abnormality; partial response (PR) as greater than 50% tumour shrinkage; stable disease (SD) as less than 50% tumour shrinkage or no change; progressive disease (PD) as an increase of at least 25%.

#### Pathological response

Many different grading systems for pathological response have been proposed (Chevallier *et al*, 1993; Sataloff *et al*, 1995; Akashi-Tanaka *et al*, 1996; Honkoop *et al*, 1998; Kuerer *et al*, 1998; Smith *et al*, 2000), but no standard method for pathological assessment after chemotherapy has been accepted. A novel simple scoring system designed to be applied in routine clinical practice was therefore devised. A consultant histopathologist (PIR) reviewed all surgical specimens for evidence of ‘histological tumour response’ defined by both (1) an apparent reduction in tumour cell : stroma ratio and (2) chemotherapy-induced cytological changes (enlarged cells with finely vacuolated cytoplasm, an enlarged vesicular nucleus with a prominent single eosinophilic nucleolus, or an enlarged hyperchromatic dense nucleus with an irregular outline). The following classification was used to score histological specimens for pathological response: pCR – no residual invasive carcinoma; pPR – residual invasive carcinoma with histological tumour response; pSD – residual invasive carcinoma with no histological tumour response. Adequate material from the resected tumour was available for pathological assessment for 99 of the 102 patients who proceeded to surgery.

### Immunohistochemical technique

Immunohistochemical staining for ER, PgR, HER-2 and Ki-67 was performed on sections of formalin-fixed paraffin-embedded tissue from core biopsies and subsequent surgical specimens mounted on poly-L-lysine slides. Slides were dewaxed and then rehydrated through graded alcohol (90, 50 and 20%). Following microwave pre-treatment in 10 mM citric acid, monoclonal mouse antibody to ER, PgR, HER-2 or Ki-67 was applied for 60 min at room temperature using the following dilutions: anti-ER – 1 : 40, anti-PgR – 1 : 60 (Novocastra Laboratories, UK); pre-diluted anti-HER-2 (Zymed Laboratories, San Francisco, CA, USA); anti-Ki-67 – 1 : 200 (DAKO Laboratories, UK). Following three rinses in tris-buffered saline (TBS) and incubation with the secondary antibody, positive brown staining was detected using a standard avidin and biotinylated horseradish peroxidase (ABC) technique with 3, 3′-diaminobenzidine (DAB) as the chromogen. Slides were then counterstained in Mayer's haematoxylin for 10 s, dehydrated in graded alcohol, mounted and scored. Positive and negative controls were performed with each stain and paired core biopsies and surgical specimens from the same patient were stained on the same run.

### Scoring methods

Immunohistochemical scoring was performed, blinded to clinical and radiological response, using the systems described below.

#### ER and PgR

A validated semi-quantitative scoring system (the ‘Allred score’) was used to assess the proportion (0=nil, 1<1/100, 2<1/10, 3<1/3, 4<2/3, 5>2/3) and intensity (0=no staining, 1=weak, 2=intermediate, 3=strong) of stained cells (Allred *et al*, 1990). A total score (range 0, 2–8) of 3 or more defined positive staining.

#### HER-2

A standard scoring system to assess both the intensity and proportion of membrane staining was used: 0 – no membrane staining or membrane staining in less than 10% of tumour cells; 1^+^ – faint barely perceptible membrane staining in more than 10% of tumour cells visible in part of the membrane only; 2^+^ – weak to moderate staining of the entire membrane in more than 10% of tumour cells; 3^+^ – strong staining of the entire membrane in more than 10% of tumour cells. Tumours scored 3+ were classed positive.

#### Ki-67

Positive and negatively stained cells were counted on a minimum of 10 randomly selected × 40 high-power fields containing representative sections of tumour. The Ki-67 proliferation index (the fraction of proliferating cells) was calculated (i.e. number of positively stained cells as a percentage of the total cell count).

### Statistical methods

Statistical analysis was carried out using JMP version 5.0. (SAS Institute Inc.). Associations between ordinal variables were assessed using *χ*^2^ analyses or the Fischer Exact Test in the case of 2 × 2 variables. Analyses involving Ki-67 as a continuous variable were investigated using ANOVA.

## RESULTS

### Response rates

A median of six cycles of chemotherapy was administered (range 2–6). At the time of analysis, 118, 94 and 99 patients were evaluable for clinical, radiological and pathological response, respectively. The overall objective clinical and radiological response rates (CR + PR) to preoperative chemotherapy were 78 and 72%, respectively. In all, 40% of patients attained a clinical CR. However, the pathological response rate was just 38%, including eight complete pathological responders (8%) among 99 assessable patients. Of the eight patients who achieved a complete pathological response, one was classified as a partial clinical and radiological responder and a further two achieved a partial radiological response; the remainder were complete responders by nonpathological criteria. Eight patients (7%) developed progressive disease during chemotherapy by clinical (five) and/or radiological (five) criteria; all the remaining 29 women had stable disease on clinical (21) and/or imaging (21) assessments (Table 2).

**Figure 1:**
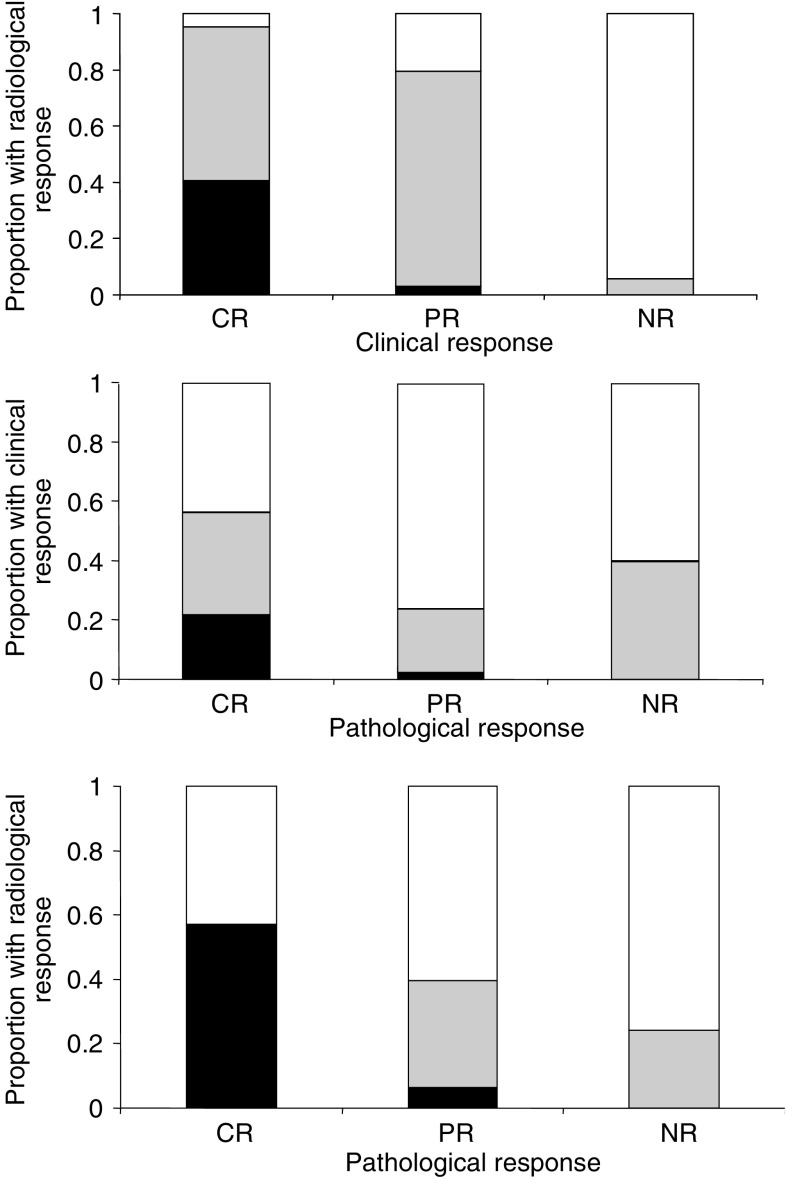
Correlation between clinical, radiological and pathological response. Solid bars represent CR; shaded bars PR and open bars NR.

Figure 1 shows the relationships between the three response end points and illustrates that significant difference in response categorisation exists depending on the chosen end point. However, when the clinical and worst radiological response data (ultrasound or mammography) were condensed to responders (CR or PR) *vs* nonresponders (SD or PD), there was almost complete agreement (98.5%) in classification of responders but a third of radiological nonresponders had a clinical response.

### Pretreatment biological parameters: inter-relationships and correlation with clinico-pathological features

The median positivity for Ki-67 was 24.9%. (range 4.1–58.5%). There was a wide range of expression patterns for ER and PgR. Overall, 63.6% of tumours were classified as ER+ and 55.1% were PgR+. The majority of ER+ (77%) and PgR+ (58%) specimens showed expression in greater than ⅓ of cells with a wide range of staining intensity; this resulted in a wide variation in the total Allred score spanning all categories from 2 to 8 (data not shown). Positive (3+) HER-2 staining was seen in 16% of tumours; 15 tumours (13%) showed weak to moderate staining (2+) deemed negative for the purpose of these analyses.

The inter-relationships between the four biological markers, pretreatment tumour size and pathological node status are shown in Table 3. There was a significant correlation between ER and PgR (*P*<0.0001): 80% of ER− tumours were also PgR−; 75% of tumours showed positivity for both steroid receptors. Higher Ki-67 proliferation indices were associated with PgR− tumours (PgR− median 28.3%, PgR+ 22.9%; *P*=0.04). A nonsignificant trend towards higher cell proliferation in pathologically node-positive tumours was observed (pathologically node negative, median Ki-67 19.6%, node positive 25.8%; *P*=0.09). There were no relationships between HER-2 expression and the other biological markers.

No relationship was seen between hormone receptor status and primary tumour size or pretreatment lymph node involvement. Ki-67 proliferation index was similar in both small and larger tumours. Clinically, node-negative patients (23 out of 61) were more likely to overexpress HER-2 than node-positive tumours (11 out of 57; *P*=0.04) (data not shown).

### Pretreatment biological parameters: correlation with treatment response

None of the pretreatment biopsy parameters correlated with any clinical, radiological or pathological response measures (Table 4). Combining the biological parameters into ‘good’ (ER+ and PgR+, low Ki-67) or ‘bad’ (ER− and PgR−, high Ki-67) risk prognostic groups also had no impact in predicting clinical response (data not shown).

### Changes in biological parameters during treatment

**Figure 2:**
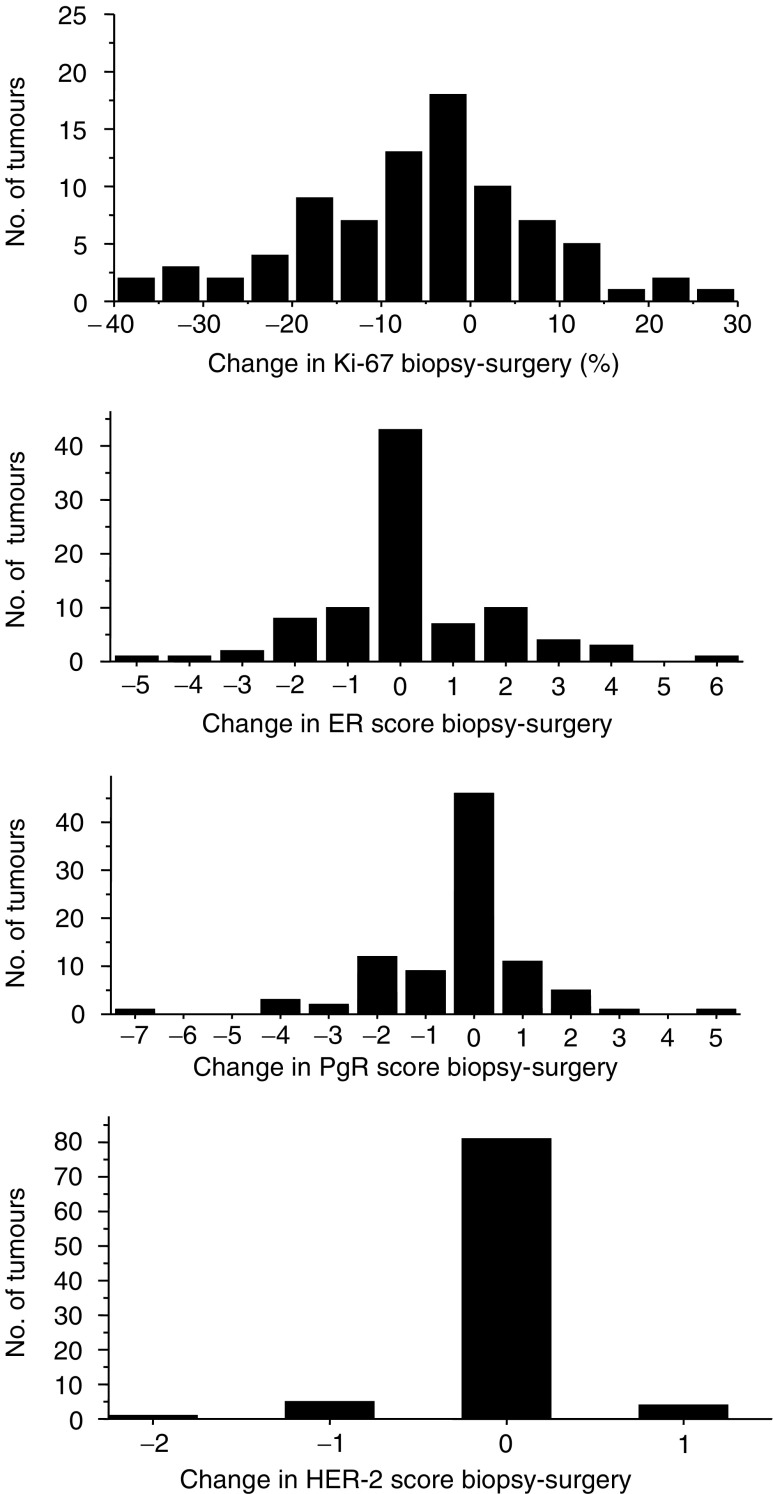
Graphical representation of change in marker status after treatment.

Figure 2 shows the changes in each biological parameter after PST. The steroid receptors showed only modest alterations during treatment. Only 13 and 25 tumours showed a shift in ER or PgR Allred score of more than 1, respectively. This resulted in three tumours changing their ER classification (positive to negative or vice versa), while seven specimens showed re-classification of their overall PgR score after chemotherapy. The median Ki-67 proliferation indices before and after treatment were 24.9 and 18.1%, respectively (*P*=0.002). Expressing the change as a function of the pretreatment Ki-67 index resulted in a median reduction in Ki-67 index after treatment of 21.2%. Of 84 evaluable patients, 47 (60%) showed little or no change (±10% of % change), 10 patients (12%) showed an increase of >50% of the original index and 24 (29%) showed a reduction of >50%. Strong or moderate HER-2 staining (2+ or 3+) at diagnosis persisted in every case after chemotherapy although HER-2 positivity (3+ cases only) changed in nine cases: five 2+ tumours were scored 3+ at operation and four 3+ tumours reverted to 2+ at surgery.

### Post-treatment biological parameters: correlation with treatment response

None of the biological markers measured in the surgical specimen correlated with any of the treatment response end points.

### Changes in biological parameters: correlation with treatment response

Changes in hormone receptor status or HER-2 did not predict treatment response by pathological, clinical or radiological criteria. Overall, there was no significant difference in the reduction in Ki-67 after treatment between responders and nonresponders when assessed by clinical, radiological or pathological criteria. However, patients with the greatest reduction in cell proliferation after chemotherapy were more likely to achieve a pathological response. Of the nine patients displaying a 75% or more fall in Ki-67 index after treatment, seven (77.8%) were pathological responders; conversely, only 20 of the remaining 75 evaluable patients (26.7%) with less extreme decreases in cell proliferation responded by histological criteria (*P*=0.004).

## DISCUSSION

The identification of reliable predictive and prognostic factors for PST in operable breast cancer remains a challenge. In the same way that ER and PgR status can predict sensitivity to endocrine therapy, reliable markers of chemosensitivity would help select those patients most likely to benefit from primary chemotherapy. More importantly, the early identification of nonresponders would allow these patients to be spared the unnecessary toxicity of ineffective chemotherapy and be offered alternative more effective treatment regimens tailored to the biological characteristics of the tumour.

Previous studies have focused on the ability to predict clinical tumour shrinkage, despite the fact that local response is a crude measure dependent on several variables including primary tumour size, oedema, necrosis and subjective variation in tumour measurements. Clinical assessment frequently overestimates tumour size (Fornage *et al*, 1987; Pain *et al*, 1992; Forouhi *et al*, 1994; Meden *et al*, 1995; Allen *et al*, 2001). Radiological assessment of maximum tumour dimensions by ultrasound or mammography is often performed to assess response and correlates better with histological tumour size than clinical measurements (Allen *et al*, 2001; Fiorentino *et al*, 2001). However, complete pathological response is the best predictor of long-term survival (Fisher *et al*, 1998). In the absence of a universally accepted optimal end point, this study therefore analysed the ability of simple biological markers to predict clinical, radiological and pathological response to treatment.

The clinical response (CR+PR) and complete pathological response (pCR) rates of 78 and 8%, respectively, seen in this series are consistent with those reported in larger randomised trials of PST (Bonadonna *et al*, 1990; Mauriac *et al*, 1991; Belembaogo *et al*, 1992; Fisher *et al*, 1997; Makris *et al*, 1998). The rates of positive staining for ER, PgR, HER-2 and Ki67 are also in accordance with expectations. Steroid hormone receptor status, HER-2 staining and Ki-67 index did not vary with tumour size or lymph node involvement, in keeping with previous observations (Bouzubar *et al*, 1989; Bottini *et al*, 1996).

In contrast with previous reports (Bonadonna *et al*, 1990; Bottini *et al*, 1996; Chang *et al*, 1999; Colleoni *et al*, 2000), clinical response rates to PST were higher in hormone receptor-positive tumours in this cohort, although the difference did not reach statistical significance. It is worthy of note that patients with hormone receptor-positive tumours in this study received chemotherapy followed by sequential adjuvant tamoxifen, rather than primary concurrent chemoendocrine treatment used in earlier reports (Makris *et al*, 1998; Chang *et al*, 1999). Consequently, any observed changes in marker status must be attributed to the direct effects of chemotherapy alone since the confounding influence of synchronous endocrine treatment has been excluded.

There is some evidence from prospective studies that HER-2 overexpression predicts sensitivity to adjuvant anthracycline chemotherapy (Muss *et al*, 1994; Clahsen *et al*, 1998; Paik *et al*, 1998; Thor *et al*, 1998) and resistance to tamoxifen (Nicholson *et al*, 1993; Leitzel *et al*, 1995; Carlomagno *et al*, 1996; Yamauchi *et al*, 1997). However, in this series, a nonsignificant trend towards poorer clinical and radiological response was seen in HER-2 overexpressors, although no difference in pathological response rates was observed. HER-2 expression was more common in ER− tumours (33 *vs* 27%) although the difference did not reach statistical significance. It should be noted that the dose of epirubicin used in this study (60 mg m^−2^) may be considered suboptimal, particularly for HER-2+ tumours. However, this was the standard dose in our institution at the time of the study.

These data confirm that no single pretreatment or postchemotherapy biological parameter predicts response by clinical, radiological or pathological criteria. With longer follow-up, it will be possible to correlate response by the three different assessment modalities with long-term survival and ultimately decide which, if any, of the methods of assessing response is best placed to act as a surrogate marker for overall survival in this cohort.

It is unlikely that any single biological marker will ever be able to reliably discriminate between responders and nonresponders, but combinations of marker expression may have greater predictive value. Intuitively, steroid receptor-negative tumours with a high proliferative rate may be expected to confer a worse prognosis. In order to test this hypothesis, we arbitrarily grouped tumours into those with a combination of ‘good’ (ER+ and PgR+, low Ki-67) or ‘bad’ (ER− and PgR− and high Ki-67) biological parameters. However, these arbitrary groups of markers also failed to discriminate between responders and nonresponders.

Although the potential effects of PST on the biological characteristics of primary breast cancers have not been widely studied, clinical decisions regarding adjuvant treatment after primary chemotherapy are frequently based on immunohistochemical analysis performed on surgical specimens pretreated with cytotoxic therapy. In this series, negative (0 or 1) and strong or moderate (2+ or 3+) HER-2 staining persisted after chemotherapy in every case. This is consistent with earlier observations that HER-2 status remains unchanged after PST (Bottini *et al*, 1996; Taucher *et al*, 2003b). In this series, some subtle changes in HER-2 staining were seen after chemotherapy. In nine of 118 cases (8%), HER-2 status changed from positive to negative or *vice versa* (five cases 2+ to 3+; four tumours 3+ to 2+). Based on these data, we recommend that patients with HER-2 scores of 0 or 1 on pretreatment biopsies do not require repeat assay after chemotherapy; however, patients with moderate or strong staining should undergo FISH analysis on a post-treatment tissue block to accurately determine the post-chemotherapy HER-2 status.

Hormone receptor expression was unchanged after treatment in the majority of tumours in this cohort, but a significant minority (10 cases, 8%) displayed a shift in ER or PgR classification at the time of surgery. In five of the 10 tumours in which changes in receptor status were described, negative staining on pretreatment core biopsies was scored positive after chemotherapy. In the remaining five cases, hormone receptor-positive core biopsies stained negative at operation. Although these observations may reflect sampling error within heterogeneous tumours, immunostaining on needle core biopsies in a previously reported series of 236 patients treated without intervening chemotherapy was highly representative of hormone receptor status in the entire resected tumour (Taucher *et al*, 2003a), suggesting that sampling error does not account for the observed hormone receptor ‘upregulation’ seen in some cases.

Some groups investigating the modulation of steroid receptor status by PST reported no significant changes in ER or PgR (Hawkins *et al*, 1990; Bottini *et al*, 1996; Schneider *et al*, 2000) after primary chemotherapy. The data presented here concur with two earlier small studies in which 10% (Lo *et al*, 1994) and 33% (Jain *et al*, 1996) of breast cancers expressed altered steroid receptor status after PST. A recently published comprehensive analysis of hormone receptor immunochemistry in 450 breast cancer patients confirmed these observations in a larger cohort and speculated on a possible hypothesis for the mechanism of changes in ER and PgR status after PST (Taucher *et al*, 2003a). Among 191 patients receiving PST, 14 of 100 ER+ tumours (14%) stained ER− after treatment, representing a statistically significant shift of ER+ to ER− status as a direct result of PST (*P*=0.02). Half of these patients (7 out of 14) were pre-menopausal, suggesting that PST may be exerting an endocrine effect by rendering women post-menopausal after chemotherapy. Our data are consistent with this hypothesis: four of the five patients displaying decreases in hormone receptor positivity after chemotherapy were premenopausal at diagnosis. Since potentially curative adjuvant hormone therapy is prescribed on the basis of hormone receptor immunostaining, we recommend that patients with ER− or PgR− status on pretreatment core biopsies should undergo repeat assay on the resected surgical specimen.

The observation that the median Ki-67 index was significantly lower after treatment in this cohort suggests that chemotherapy is exerting a true anti-proliferative effect on tumours *in vivo*. Both primary endocrine (Clarke *et al*, 1993) and cytotoxic treatments (Daidone *et al*, 1991; Gardin *et al*, 1994) inhibit tumour growth. Tumour cell proliferation has been shown to be a prognostic marker in some reports (Remvikos *et al*, 1989; Bonetti *et al*, 1996; Chevillard *et al*, 1996), but data on its predictive value are conflicting. S-phase fraction, proliferating cell nuclear antigen and thymidine labelling index predict overall survival (Clayton, 1991; Fisher *et al*, 1991; Merkel *et al*, 1993) and response (Collecchi *et al*, 1996; Bottini *et al*, 1998; Nishimura *et al*, 2002), but not disease-free survival (Bianchi *et al*, 1993; Pisansky *et al*, 1993; Tahan *et al*, 1993) in patients treated with primary surgery or chemotherapy.

Baseline Ki-67 index did not predict clinical response in this cohort of patients. This is consistent with other studies (Bonadonna *et al*, 1990; Makris *et al*, 1997), but in contrast to data from other groups, which suggested that S-phase fraction (Remvikos *et al*, 1989), Ki-67 index (Bottini *et al*, 1996) or thymidine labelling index (Collecchi *et al*, 1998) predict clinical tumour shrinkage. To the best of our knowledge, pathological response has not been formally assessed in previous studies analysing the predictive value of Ki-67 before and after PST. In this study, pretreatment cell proliferation failed to predict pathological response. This suggests that chemosensitivity is not simply a function of the baseline rate of tumour cell proliferation, and supports the theory that some rapidly proliferating tumours are inherently chemoresistant. However, tumours that displayed a large reduction in Ki-67 index after chemotherapy were more likely to achieve a pathological response: 78% of patients (seven out of nine) with falls of >75% in Ki-67 after treatment were pathological responders compared to 20 out of 75 (27%) of those with less extreme falls in Ki-67. These findings have not been previously reported. Intuitively, this observation suggests that the antiproliferative and proapoptotic effects of chemotherapy are indeed translated into a measurable histological response.

Like many studies in this field, this work is hindered by the fact that complete pathological response and clinical progression – the two scenarios that clinicians would most like to be able to predict – are relatively uncommon events. The majority of patients achieve a PR by clinical or radiological criteria and nearly two-thirds showed SD by pathological assessment. Consequently, large studies are required if reliable predictive markers are to be identified.

In conclusion, the baseline biological characteristics of primary breast tumours analysed in this study did not predict response to treatment by any modality of assessment. However, patients with the greatest reductions in tumour cell proliferation on completion of chemotherapy were most likely to achieve a pathological response. With longer follow-up, it will be possible to establish if chemotherapy-induced changes in cell proliferation have prognostic value, and to determine whether the pathological response scoring system described here can be used as a surrogate end point for long-term clinical outcome.

Novel markers that relate to the actions of chemotherapy drugs are needed if reliable predictive markers are to be identified. One such example is topoisomerase II*α*, a molecular target for anthracyclines. A recent study showed that strong topoisomerase II*α* staining is an independent predictor of clinical tumour regression (MacGrogan *et al*, 2003); a confirmatory study is underway on this data set. In the meantime, it is imperative that large ongoing randomised clinical trials of new PST regimens encourage recruitment into parallel biological marker studies so that more powerful data sets can continue the search for favourable and unfavourable biological profiles that may ultimately help clinicians individualise treatments. Meanwhile, the ability to study the patterns of expression of thousands of candidate genes simultaneously using new micro-array technologies (Perou *et al*, 2000; Chang *et al*, 2003) may rapidly surpass retrospective analyses using immunohistochemistry in the continuing search for predictive and prognostic factors.

## Figures and Tables

**Table 1:** Patient characteristics

	***n***	**%**
Age: median (years)	48	
Age: range (years)	25–78	
		
*Menstrual status*		
Pre	69	59
Peri	19	16
Post	30	25
		
*Clinical TNM stage*		
T2	61	52
T3	42	36
T4	15	12
		
*Clinical node status*		
N0	61	52
N1	57	48
		
*Tumour grade*		
G1	11	14
G2	35	44
G3	33	42
		
*Breast*		
Right	64	54
Left	53	45
Bilateral	1	1
		
*Chemotherapy regimen*		
FEC	91	77
AC	9	8
CAF	2	2
ECF	2	2
MMM	14	12

**Table 2:** Response rates [n (%)] by method of assessment

**Assessment**	***n***	**ORR^a^**	**CR**	**PR**	**SD**	**PD**
Clinical	118	92 (78%)	47 (40%)	45 (38%)	21 (18%)	5 (4%)
Radiological	94	68 (72%)	18 (19%)	50 (53%)	21 (22%)	5 (5%)
Pathological	99	38 (38%)	8 (8%)	30 (30%)	61 (62%)	—

a ORR: overall response rate=CR+PR.

**Table 3:** Relationship between clinical and biological variables

	**T2**	**T3/4**	***P***
ER+	39/61 (64.0%)	36/57 (63.2%)	NS
PgR+	34/61 (55.7%)	31/57 (54.4%)	NS
HER-2+	21/61 (34.4%)	13/57 (22.8%)	NS
Ki-67 median	25.8%	23.1%	NS
			
	**ER−**	**ER+**	
PgR+	9/43 (20.9%)	56/75 (74.7%)	<0.0001
HER-2+	14/43 (32.6%)	20/75 (26.7%)	NS
Ki-67 median	28.6%	23.1%	NS
			
	**HER-2−**	**HER-2+**	
ER+	55/84 (65.5%)	20/34 (58.8%)	NS
PgR+	49/84 (58.3%)	16/34 (47.1%)	NS
Ki-67 median	25.4%	23.5%	NS
			
	**PgR−**	**PgR+**	
Ki-67 median	28.3%	22.9%	0.04

**Table 4:** Response rates (CR + PR) by biopsy marker status

	**Clinical response (%)**	**Radiological response (%)**	**Pathological response (%)**
All patients	78	72	38
ER+	81	73	34
ER−	72	71	46
*P*-value	0.25	0.81	0.29
			
PgR+	83	78	31
PgR−	72	65	47
*P*-value	0.18	0.17	0.15
			
HER-2+	71	62	44
HER-2−	81	77	36
*P*-value	0.23	0.15	0.51
			
Ki-67 high	73	74	39
Ki-67 low	81	70	38
*P*-value	0.38	0.81	1.0

Each parameter was dichotomised as described in the text or above and below the median value for Ki-67.
